# Semi-supervised vision transformer with adaptive token sampling for breast cancer classification

**DOI:** 10.3389/fphar.2022.929755

**Published:** 2022-07-22

**Authors:** Wei Wang, Ran Jiang, Ning Cui, Qian Li, Feng Yuan, Zhifeng Xiao

**Affiliations:** ^1^ Department of Breast Surgery, Hubei Provincial Clinical Research Center for Breast Cancer, Hubei Cancer Hospital, Tongji Medical College, Huazhong University of Science and Technology, Wuhan, Hubei, China; ^2^ Department of Thyroid and Breast Surgery, Maternal and Child Health Hospital of Hubei Province, Wuhan, Hubei, China; ^3^ Department of Ultrasound, Hubei Cancer Hospital, Tongji Medical College, Huazhong University of Science and Technology, Wuhan, Hubei, China; ^4^ School of Engineering,Penn State Erie, The Behrend College, Erie, PA, United States

**Keywords:** semi-supervised learning, breast cancer detection, vision transformer, adaptive token sampling, data enhancement

## Abstract

Various imaging techniques combined with machine learning (ML) models have been used to build computer-aided diagnosis (CAD) systems for breast cancer (BC) detection and classification. The rise of deep learning models in recent years, represented by convolutional neural network (CNN) models, has pushed the accuracy of ML-based CAD systems to a new level that is comparable to human experts. Existing studies have explored the usage of a wide spectrum of CNN models for BC detection, and supervised learning has been the mainstream. In this study, we propose a semi-supervised learning framework based on the Vision Transformer (ViT). The ViT is a model that has been validated to outperform CNN models on numerous classification benchmarks but its application in BC detection has been rare. The proposed method offers a custom semi-supervised learning procedure that unifies both supervised and consistency training to enhance the robustness of the model. In addition, the method uses an adaptive token sampling technique that can strategically sample the most significant tokens from the input image, leading to an effective performance gain. We validate our method on two datasets with ultrasound and histopathology images. Results demonstrate that our method can consistently outperform the CNN baselines for both learning tasks. The code repository of the project is available at https://github.com/FeiYee/Breast-area-TWO.

## 1 Introduction

Breast cancer (BC) has been the most common cancer type for women. The 2020 report of the World Cancer Research Fund shows that there were more than 2 million newly diagnosed BC cases in 2018 (Bray et al., 2018). Such worrying numbers highlight the significance of properly using present technological advancements to undertake efficient BC detection in its early stage. In particular, a recent development in artificial intelligence (AI) that explores the usage of deep learning models in a wide spectrum of health care applications presents a promising direction toward building a more effective computer-aided diagnosis (CAD) system for BC detection (Hu et al., 2020; Mewada et al., 2020; Moon et al., 2020; Boumaraf et al., 2021; Eroğlu et al., 2021; Mishra et al., 2021).

A variety of imaging techniques can be used for BC detection and diagnosis, including X-rays (mammograms) (Abdelrahman et al., 2021), ultrasound (sonography) (Moon et al., 2020; Mishra et al., 2021), thermography (Singh and Singh, 2020), magnetic resonance imaging (MRI) (Mann et al., 2019), and histopathology imaging (Benhammou et al., 2020). Ultrasound has been a widely adopted, low-cost, non-invasive, and non-radioactive imaging modality in the procedure of BC diagnosis and is usually followed by histopathological analysis. The latter applies biopsy techniques to collect cell/tissue samples that are placed on a microscope slide and then stained for microscopic examination. With a high degree of confidence, histopathological diagnosis has become the gold standard for almost all cancer types (Das et al., 2020). However, in spite of the usage of various imaging modalities, it requires radiologists or pathologists to perform a visual inspection, which is time-consuming and in need of a high degree of radiological/pathological expertise. In addition, it has been shown by several studies that a high percentage of inter-observer variability exists when the same set of images are read by different experts (Kaushal et al., 2019). An AI-powered system has the potential to eliminate this assessment discrepancy caused by different experiences, analytical methodology, and knowledge between human beings, providing a more accurate diagnostic result to support clinical decision-making (Hamed et al., 2020).

Recent advances in AI, especially in deep learning, have been extensively investigated in the health care industry (Beam and Kohane, 2018; Li and Xiao, 2022; Qu and Xiao, 2022). The number of use cases of deep learning in BC detection has also been increasing (Hamed et al., 2020). Our literature investigation shows that prior efforts in breast cancer image classification share two common characteristics. First, the learning models are mostly based on the convolutional neural network (CNN), including existing deep CNN architectures, custom CNNs, and hybrid models with a CNN as a component. Despite the effectiveness of CNN-based classification models, recent advances have witnessed the rise of a novel vision model, namely, the Vision Transformer (ViT) (Dosovitskiy et al., 2020), which has been shown to be more accurate in multiple public benchmarks. Few studies have investigated the usage of the ViT in BC detection (Gheflati and Rivaz, 2021), and the potential of the ViT has not been fully explored in this area. Second, most existing studies are based on supervised learning, which requires a full annotation for all image samples in the dataset. The procedure of annotation is time-consuming and requires domain expertise. Semi-supervised learning (SSL) (Van Engelen and Hoos, 2020), on the other hand, only requires annotation on a small subset of training data and combines a larger subset of unlabeled data during training. SSL can effectively reduce the efforts of annotation. However, SSL has not been extensively used in present studies of BC detection.

Our study aims to address these methodological gaps. Specifically, we propose a ViT-based BC classification learning pipeline that combines both supervised learning and SSL. We use an adaptive token sampling (ATS) technique (Fayyaz et al., 2021) that allows the original ViT model to dynamically choose the most critical image tokens. Moreover, we present a custom consistency training (CT) strategy (Xie et al., 2020) to unify supervised and unsupervised learning with image augmentation. The CT-based SSL, when combined with an ATS-ViT (namely, ViT with ATS), can effectively boost the model performance. The proposed method has been validated on two datasets, including the dataset of breast ultrasound images (BUSI) (Al-Dhabyani et al., 2020) and the Breast Cancer Histopathological Image Classification (BreakHis) dataset (Spanhol et al., 2016). The results of our method have been promising and superior compared to the CNN models. The project is released under the MIT License and is available at https://github.com/FeiYee/Breast-area-TWO.

The rest of this study is organized as follows. We provide a literature review for relevant studies in Section 2. Section 3 describes the datasets used in this study and the details of the proposed model. In Section 4, several experiments are conducted to evaluate the effectiveness of the proposed model. Finally, in Section 5, we conclude the study and provide future work.

## 2 Related work

This section reviews the prior studies in two aspects, including DNN-based BC detection methods and SSL applied in biomedical image classification.

### 2.1 Deep neural network-based breast cancer detection

Numerous existing and custom deep CNN models have been used on both ultrasound and histopathology images for breast tumor classification. Compared to feature-based learning models that require hand-crafted features (Mishra et al., 2021), deep neural network (DNN) models such as CNNs can learn discriminative patterns with automatically extracted features to represent an image sample (Li et al., 2021). For ultrasound imaging, Masud et al. (2020) proposed a custom CNN model compared with several existing CNN models, including AlexNet (Kri zhevsky et al., 2012), Darknet19 (Redmon et al., 2016), GoogleNet (Szegedy et al., 2015), MobileNet (Howard et al., 2017), ResNet18 (He et al., 2016), ResNet50, VGG16 (Simonyan and Zisserman, 2014), and Xception (Chollet, 2017). In addition to single models, ensemble learning has also been used. Moon et al. (2020) aggregated three CNN models, including VGGNet, ResNet, and DenseNet (Huang et al., 2017) by fusing the image representations. Similarly, Eroğlu et al. (2021) adopted a concatenation of features generated by Alexnet, MobilenetV2 (Sa ndler et al., 2018), and Resnet50, followed by a Minimum Redundancy Maximum Relevance-based feature selection strategy to choose a set of the most valuable features that were used to train a feature-based classifier [e.g., support vector machine (SVM) (Pisner and Schnyer, 2020), k-nearest neighbors (KNNs) (Peterson, 2009)]. As for histopathology imaging, prior studies have adopted CNN models with improvements in several aspects. Alom et al. (2019) proposed an Inception Recurrent Residual Convolutional Neural Network (IRRCNN) to combine the predictive power of the recurrent CNN, ResNet, and the Inception network. Wang et al. developed FE-BkCapsNet that integrates the CNN and CapsNet (Sabour et al., 2017) with deep feature fusion and enhanced routing. Mewada et al. (2020) proposed the use of both the spatial features of a CNN and the spectral features of a wavelet transform to address the convergence issue during training. In addition to the improvements in models, novel training strategies have also been developed. Boumaraf et al. (2021) used a block-wise fine-tuning method, allowing the last few residual blocks in the CNN to be more domain-specific. Despite the extensive studies of DNN-based models for BC detection, other model types have not been fully explored. The ViT, as a recently developed and highlighted vision model, has received significant attention in a wide range of tasks. It is desirable to validate the effect of the ViT in imaging-based BC detection. Our study is such an attempt.

### 2.2 Semi-supervised learning-based biomedical image classification

SSL has been an effective training technique to reduce the number of training examples required for a fully supervised learning procedure. Obtaining a data point in the biomedical domain could be time-consuming, especially in the field of cancer research, where it could take months or even years to determine a patient’s final status (Zemmal et al., 2016). Thus, prior studies have adopted SSL to use the unlabeled data. Zemmal et al. (2016) adopted a Semi-Supervised Support Vector Machine (S3VM) with hand-crafted features for BC detection. Jaiswal et al. (2019) used pseudo labels on the PatchCamelyon-level to detect metastasized cancer cells in histopathology diagnosis.Shi and Zhang (2011) used low-density separation, an SSL method, to conduct gene expression-based outcome prediction for cancer recurrence. Ma and Zhang (2018) developed an SSL model that combines affinity network fusion and a neural network to implement few-shot learning, significantly improving the model’s learning ability with fewer training data. Other applications of SSL include cancer survival analysis (Liang et al., 2016), skin cancer diagnosis (Masood et al., 2015), bladder cancer grading (Wenger et al., 2022), and colorectal cancer detection (Yu et al., 2021). To our best knowledge, prior studies have not explored CT for BC detection, and our research aims to fill this gap.

## 3 Materials and methods

### 3.1 Dataset

Two datasets are used to validate the proposed method, including the dataset of breast ultrasound images (BUSI) (Al-Dhabyani et al., 2020) and the Breast Cancer Histopathological Image Classification (BreakHis) dataset (Spanhol et al., 2016) that represent non-invasive and invasive BC detection methods, respectively. Also, the choice of these two datasets allows our model to be trained and validated using images from diverse sources, which can be used to evaluate a model’s robustness.

#### 3.1.1 Breast ultrasound images dataset

**FIGURE 1:**
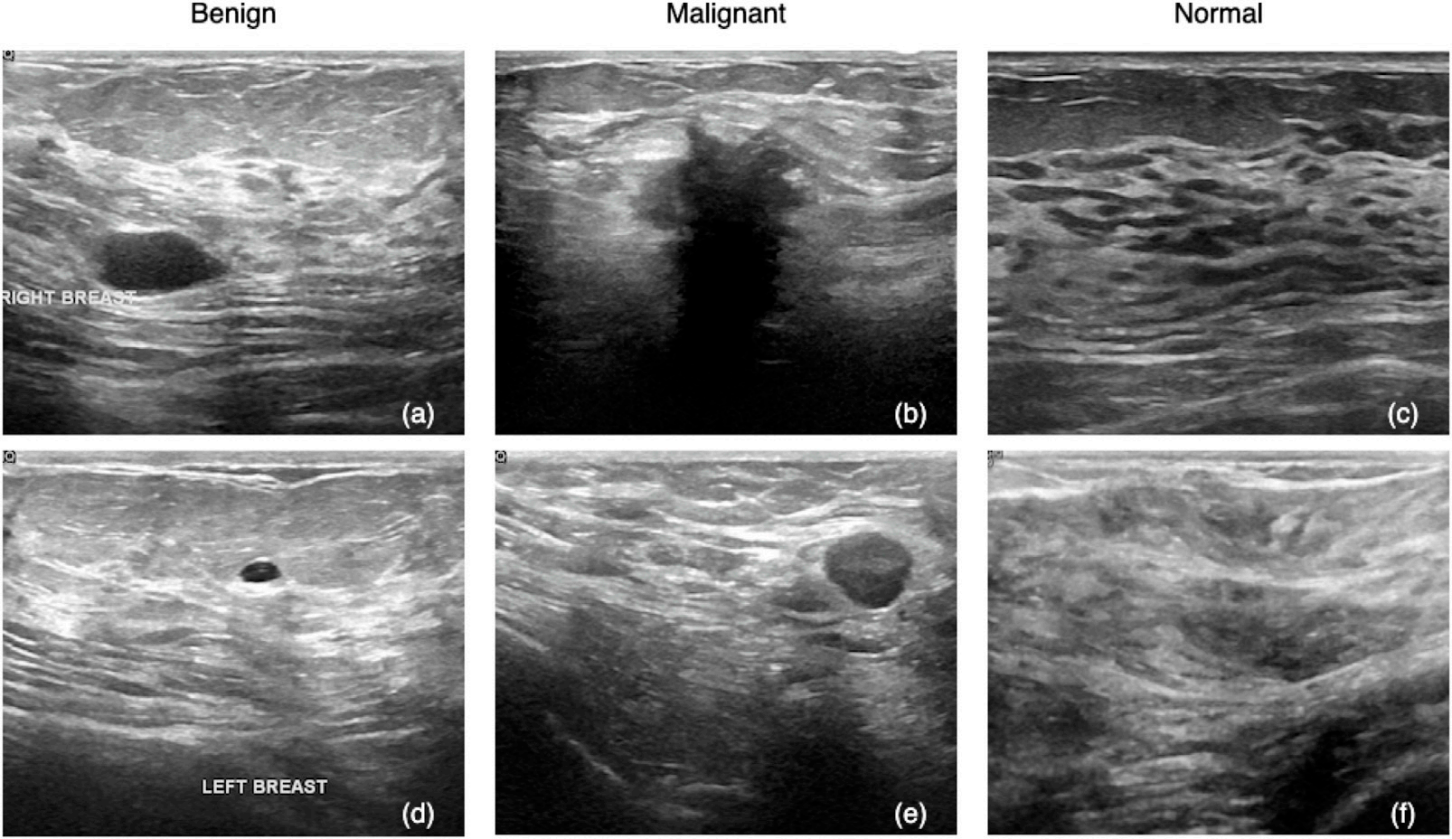
BUSI samples: **(A,D)** are benign tumor samples, **(B,E)** are malignant, and **(C,F)** are normal.

Table 1 shows the three classes of BUSI and the number of image samples for each class. Typically, ultrasound images are in grayscale. The images were gathered at the Baheya hospital, saved in DICOM format, and converted to PNG format afterward. Data collection and annotation took around 1 year to complete. The total number of images acquired at the start of the project was 1,100, which decreased to 780 after preprocessing to eliminate images with unimportant information. The LOGIQ E9 and the LOGIQ E9 Agile ultrasound systems were used in the scanning procedure, producing images with a resolution of 1280 × 1024. Figure 1 shows two example samples per class, totaling six samples, in which (a) and (d) are benign, (b) and (e) are malignant, and (c) and (f) are normal. An experienced radiologist reads an ultrasound image based on a set of standard criteria that involve mass size, echo nodule, tumor borders and morphology, calcification, blood flow, and so on. These criteria can be regarded as discriminative features allowing a trained human being to determine the class of an image. Traditional feature-based models encode these criteria into hand-crafted features to represent an image, while DNN-based models can automatically extract discriminative patterns and yield a higher accuracy (Shaheen et al., 2016; Han et al., 2017).

**TABLE 1:** Three classes in the DBUI dataset.

Class	# Images per class
Benign	487
Malignant	210
Normal	133
Total	780

#### 3.1.2 BreakHis dataset

The BreakHis dataset contains 7,909 microscopic images of breast tumor tissue, including 2,480 benign and 5,429 malignant samples, collected from 82 patients by the P&D Laboratory–Pathological Anatomy and Cytopathology, Parana, Brazil. These images are with four magnifying factors, namely, ×40, ×100, ×200, and ×400. All of the samples are of 700 × 460 pixels with 3-channel RGB and 8-bit depth in each channel, stored in PNG format. A histologically benign sample does not meet any malignancy criteria such as mitosis, basement membranes disruption, metastasize, etc. In other words, benign tumors grow slowly and stay localized. On the contrary, the malignant ones have locally invasive lesions that can disrupt adjacent structures and lead to metastasis to distant sites of the human body. Table 2 shows a stats summary of the BreakHis dataset.

**TABLE 2:** Stats of the BreakHis dataset.

Magnification	Benign	Malignant	Total
x40	625	1,370	1,995
x100	644	1,437	2,081
x200	623	1,390	2,013
x400	588	1,232	1,820
Total	2,480	5,429	7,909
# Patients	24	58	82

**FIGURE 2:**
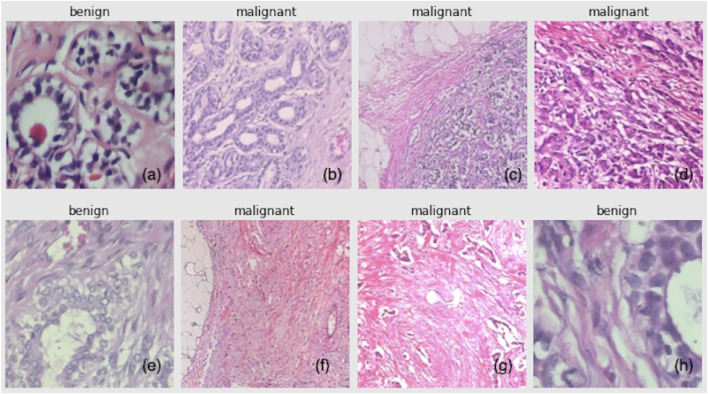
BreakHis samples: **(A,E,H)** are benign, and **(B**–**D,F,G)** are malignant.

The breast tissue slides are imaged digitally using an Olympus BX-50 system microscope equipped with a 3.3x relay lens and a Samsung SCC-131AN digital color camera. The collected slides are then stained with hematoxylin and eosin (HE). The samples are obtained through surgical (open) biopsy (SOB), which is then processed for histological examination and labeled by pathologists from the P&D Laboratory. The standard paraffin method, which is widely used in clinical routine, was used in the preparation of the samples in this study. The primary purpose is to keep the original tissue structure and molecular composition, which allows it to be observed under a light microscope in its natural state. After staining, the anatomopathologists visually examine the tissue samples with a microscope to determine whether or not there are any cancerous lesions present in each slide. Experienced pathologists make the final diagnosis in each case, which is then confirmed by additional tests such as immunohistochemistry (IHC) analysis. Figure 2 shows a set of samples from the BreakHis dataset, in which the subfigures (a), (e), and (h) are benign samples, and the rest are all malignant.

### 3.2 Overview of the learning framework

**FIGURE 3:**
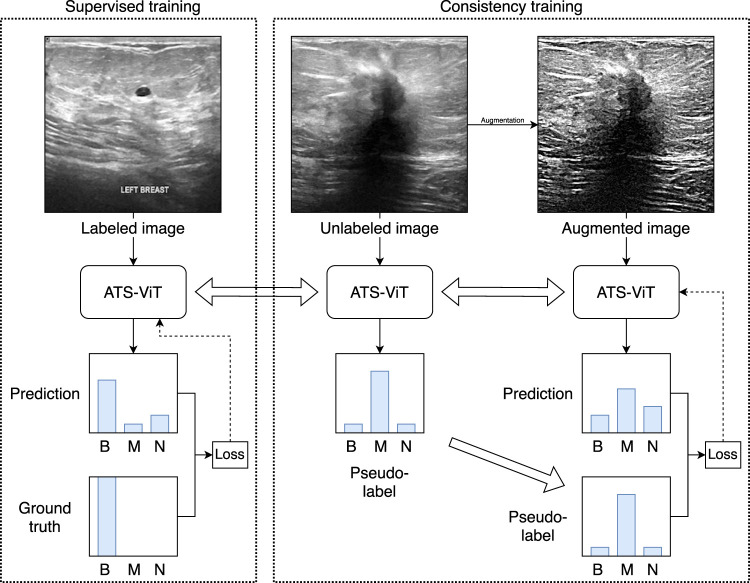
Overview of the proposed learning framework. The framework comprises supervised and consistency training unified *via* an end-to-end training procedure. For simplicity, the figure only uses image samples from the BUSI dataset. The method has been validated on both datasets used in this study.

Figure 3 shows the overall workflow of the proposed method. The core model to be trained is the ATS-ViT. The training procedure comprises two parts, namely, supervised and consistency training. The former aims to improve the model’s predictive ability, and the latter improves its generalization. Both parts are unified *via* an end-to-end training procedure (described in Algorithm 1). It should be noted that the parameters of the ATS-ViT are shared across both parts of training. Also, three types of losses are combined to guide the optimization of the neural network via gradient descent. The training details are covered in Subsection 3.6.

### 3.3 Transformer

A transformer (Vaswani et al., 2017) is a neural architecture that uses an attention mechanism to mine and capture the semantic meanings and relations among the input tokens for sequential modeling problems. One of the benefits of the transformer is that it allows parallelization since tokens passing through its architecture can be processed independently rather than sequentially, presenting a unique advantage over recurrent models such as long short term memory (LSTM) (Kim et al., 2016) and recurrent gated unit (GRU) (Chung et al., 2014). The transformer was originally designed for machine translation in natural language processing (NLP) and showed superior performance. Moreover, recent advances have explored applications of the transformer in a wide spectrum of NLP tasks and developed a rich set of pre-training techniques, making it one of the most influential works in AI in the past 5 years.

A transformer adopts an encoder-decoder structure. The encoder module comprises a stack of transformer encoders; similarly, the decoder module is a stack of transformer decoders. Each transformer encoder includes a self-attention layer with multiple attention heads to capture the semantic interaction among the input tokens. Specifically, each attention head calculates a tensor of scores to express how each token is affected (attended) by every other token. The outputs of these attention heads are aggregated, normalized, and passed to a feed-forward layer to generate a set of embeddings, which are the output of the present encoder. The subsequent encoder takes as input the embeddings generated from its previous encoder and repeats the process. A transformer decoder, on the other hand, comprises three layers, including a multi-head self-attention layer, an encoder-decoder attention layer, and a feed-forward layer. At each time step, a transformer decoder takes as input two intermediate tensors generated by the last encoder layer, the embeddings from its previous decoder (it would be the output of the decoder module at the previous time step for the first decoder); these data are fed through a stack of decoders, followed by a linear and a softmax layer to produce the prediction result.

### 3.4 Vision transformer

The wide success of a transformer in NLP tasks inspired researchers to explore its potential in computer vision. The ViT has been one of the first efforts. The ViT adopts the same structure as the original transformer with the following changes to the input. An image is chunked into a set of image patches to meet the input requirement of the transformer. The so-called image patch embedding operation is essentially a linear transformation, that is, a fully connected layer. Specifically, if an input image of size *H* × *W* × *C* is split into *N* patches (i.e., tokens), each of size *P* × *P* × *C*, then we can determine that $N=\frac{HW}{P^{2}}$ . Then, each patch is spread out into a vector of size *D*. Thus, the input is transformed into a 2D tensor of size *N* × *D*. In addition, a special [CLS] token is inserted into the first position of the token sequence to encode the information used for classification. This strategy has been commonly seen in other pre-training strategies such as the Bidirectional Encoder Representations from Transformers (BERT) (Devlin et al., 2018). Furthermore, to maintain the relative position relationship between different patches, a position encoding vector is added to each patch embedding, generating a token embedding used by the first layer of the transformer encoder.

### 3.5 Adaptive token sampler

**FIGURE 4:**
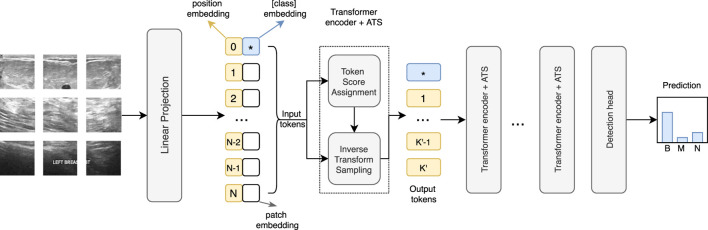
Architecture of the ATS-ViT. The ATS module can be integrated into each transformer block to perform two steps, including token score assignment and inverse transform sampling. The ATS can identify the most informative tokens that are passed to the subsequent layers, effectively reducing the computational cost and improving the classification accuracy.

The ViT is computationally expensive since the computing cost rises quadratically with the number of tokens. CNNs reduce the resolution inside the network with different pooling operations. However, because the tokens are permutation invariant, using pooling in the ViT is not feasible. Thus, we adopt an adaptive token sampler (ATS), a technique that allows the model to dynamically choose significant tokens from the input tokens to reduce computational cost. Figure 4 shows the network structure of ViT with ATS.

An ATS works by assigning a score to each of the *N* input tokens to determine which ones to keep. The score indicates a token’s contribution to the final prediction. Let *K* be the maximum number of retained tokens, and a sampling strategy is adopted as follows. Let K , Q , and V be the query, key, and value vectors, respectively, in the standard self-attention layer of the transformer. The attention matrix A can be computed *via* Eq. 1.

$$A=Softmax\left(\frac{QK^{⊤}}{\sqrt{d}}\right).$$ (1)

Thus, A is (*N* + 1) × (*N* + 1) (with the [CLS] token counted) and sums up to 1 after the softmax operation. The output tokens, before sampling, are given by Eq. 2.

O=AV. (2)

Let $A_{i,j}$ denote the element at row *i* and column *j* in A , the significance score of token *j* can be calculated by Eq. 3.

$$S_{j}=\frac{A_{1,j}\|V_{j}\|}{\sum_{i=2}A_{1,i}\|V_{i}\|}.$$ (3)

Only the first row of the attention matrix A is used since each element $A_{1,j}$ represents the importance of token *j* to token 1, namely, the [cls] token. With a significance score calculated for each input token, the inverse transform sampling strategy is used for token sampling. First, the cumulative distribution function of S can be calculated *via* Eq. (4).

$$CDF_{i}=\overset{j=i}{\underset{j=2}{\sum }}S_{j}.$$ (4)

It is noted that the first token is excluded since it is used to encode the classification information, and thus, is not needed for the calculation of the CDF. The sampling function, denoted by ϒ(*k*), can now be obtained via the inverse function of the CDF, which is given by Eq. 5.

$$ϒ\left(k\right)=CDF^{-1}\left(k\right).$$ (5)

To obtain *K*′ samples (*K*′ ≤ *K*), ϒ(⋅) is run *K*′ times from uniform distribution *U*[0, 1], which generates *K*′ real numbers that are rounded to the nearest integers and used as the sampling indices. The selected *K*′ output tokens should carry more informative patterns and are passed to the next transformer block.

### 3.6 Semi-supervised learning

SSL is a training paradigm that explores both labeled and unlabeled data to enhance the robustness of a model. Also, SSL is a popular strategy when the number of training samples is limited because of high annotation costs. In this study, we assume that similar images should belong to the same class, which is referred to as the smoothness assumption and has been adopted by many SSL training systems (Chen and Wang, 2010). CT is a typical SSL method used in prior studies (Xie et al., 2020; Lee and Cho, 2021). CT allows a model to be trained to yield consistent results for an image and its augmented versions with various perturbations such as crop, contrast, flip, jittering, etc. The proposed CT method is described in detail as follows.

First, we divide the original training set *X* into two sets *X*_*l*_ and *X*_*u*_, treated as labeled and unlabeled datasets during CT, respectively. Second, a set of image augmentation algorithms ${\left\{h_{i}\right\}}_{i=1}^{m}$ are defined. An unlabeled sample *x*_*u*_ is fed into algorithm *h*_*i*_ to generate an augmented image denoted by *z*_*u*,*i*_. Let **F** denote the ViT model. The training objective of our SSL algorithm is three-fold.

• First, the supervised loss should be minimized to improve the predictive ability of model **F**. For our study, the binary cross-entropy loss is used, denoted by *L*_*CE*_. For a batch of *m* labeled samples ${\left\{\left(x_{l},y_{l}\right)\right\}}_{l=1}^{m}$ , we can calculate *L*_*CE*_ based on Eq. 6

$$L_{CE}=-\frac{1}{m}\overset{m}{\underset{l=1}{\sum }}y_{l}\log F\left(x_{l}\right).$$ (6)

• Second, the pseudo-label loss should be minimized to encourage the model to produce consistent results for an image and its augmented versions with perturbations. For each image *x*_*u*_ in a batch of *m* unlabeled data, a random augmentation algorithm is selected from ${\left\{h_{i}\right\}}_{i=1}^{m}$ and applied to the image *x*_*u*_ to generate an augmented image *z*_*u*_. Let **F**(*x*_*u*_) be a pseudo-label, and we can then calculate pseudo-label loss using the mean squared error based on Eq. 7.

$$L_{\mathrm{MSE}}=\frac{1}{m}\overset{m}{\underset{u=1}{\sum }}{\left(F\left(x_{u}\right)-F\left(z_{u}\right)\right)}^{2}.$$ (7)

• Last, to ensure the consistency of the whole process, we also need to measure the intermediate result of unlabeled data and its augmented version, and since the intermediate result of the ViT is a one-dimensional sequence, we use Earth Mover’s distance (Rubner et al., 2000), noted as *L*_*EM*_, which is used to describe the degree of similarity of two distributions. Given two sets of distributions *p*_1_, *p*_2_….*p*_*m*_ and *q*_1_, *q*_2_….*q*_*m*_, we need to find a way to arrange *q* in such a way that the EML loss is minimized. The loss can be given by Eq. 8.

$$L_{EM}\left(p,q\right)=\underset{q\in Q}{\min}\overset{m}{\underset{i}{\sum }}l\left(q_{i},p_{i}\right),$$ (8)

where *Q* is the set of all possible permutations of *q* and *l* stands for the measurement, here, we choose it as L2 loss.

Aggregating the three aforementioned individual losses yields the following overall loss function, which is our final optimization objective.

$$L=L_{CE}+L_{\mathrm{MSE}}+L_{EM}.$$ (9)

When we ask the model to obtain similar features for data before and after adding multiple join perturbations, we can force the model to learn what does not change with perturbation, and the information that remains constant before and after perturbation is more relevant to the classification result, and such a strategy will lead to stronger generalization ability. Therefore, we can confirm that combining data augmentation strategies with semi-supervised learning can give better results.

Algorithm 1 SSL algorithm.

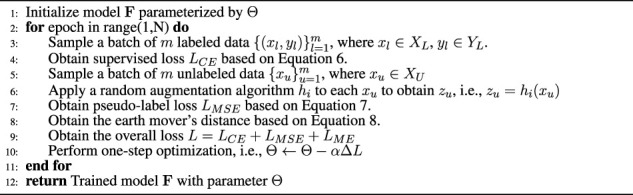

## 4 Results

Codes in this study have been written in Python 3.6.10 and using PyTorch 1.8.0 as the deep learning framework. All experiments were run on a workstation with a Windows 10 operating system, an i7-10875h CPU, and an Nvidia GTX2080TI 12G graphic card.

### 4.1 Evaluation metrics

Since the classes for both datasets are imbalanced, accuracy (Acc) is not sufficient to reflect the true performance of a model. Therefore, in addition to ACC, we also use precision (Pre), recall (Rec), and F1 scores for performance evaluation. These indicators are defined in Eqs 10–13.

$$Acc=\frac{TP+TN}{TP+TN+FP+FN},$$ (10)

$$Pre=\frac{TP}{TP+FP}\times ,$$ (11)

$$Rec=\frac{TP}{TP+FN}\times ,$$ (12)

$$F1=2\times \frac{Pre\times Rec}{Pre+Rec},$$ (13)

where TP, TN, FP, and FN refer to the number of true positives, true negatives, false positives, and false negatives, respectively. Pre reflects the ratio of false alarms. The higher the pre, the fewer false alarms the model has. Meanwhile, Rec reflects the quantity of missed positive samples. In other words, the higher the Rec, the fewer positive samples that have been missed. F1 represents the harmonic mean of Pre and Rec, presenting a more suitable metric than Acc for a classification task with an imbalanced dataset.

### 4.2 Baselines

Four models have been chosen as the baselines in this study, namely, the VGG16, ResNet101, DenseNet201, and ViT. All four models have been extensively used in a variety of image classification tasks and served as solid baselines.

• The VGG16 network comprises a sequence of five blocks, each with two or three convolutional layers for feature extraction, followed by a pooling layer for downscale sampling. The last block is further followed by three fully connected layers and a softmax layer to generate a normalized vector as the prediction result. The VGG neural architecture extensively uses small (3 × 3) convolutional filters, which is the basis for building a deep and accurate network.
• The ResNet neural architecture stacks a sequence of residual blocks, each of which facilitates the learning of an identity function *via* a shortcut connection by feeding the input of a block directly into the output. This way, an identify function can be easily learned, allowing a network with more layers to be trained more effectively without diminishing returns. ResNet101 contains a series of repeated residual blocks followed by a dense and a softmax layer, with a total of 101 layers.
• DenseNet is a variant of ResNet with two differences. First, DenseNet uses a concatenation instead of a summation (used in ResNet) to aggregate the layer output and the shortcut data within each block. Second, DenseNet introduces a transition layer placed between two dense blocks. Each transition layer comprises a 1 × 1 convolutional layer and an average pooling layer with a stride of two to control the model complexity.
• The ViT has been covered in Section 3.4.

### 4.3 Training setting

The main hyperparameters used for training are shown in Table 3. We adopted Adam as the optimizer with a learning rate of 2e-5. We set eps = 1e-08 to prevent the denominator from being 0. A batch size of 64 was chosen. The loss function was the binary cross entropy with logits. All evaluated models were trained with 300 epochs. For the ViT, each input image was re-scaled to a fixed size of 256 × 256 and split into 16 patches. The ViT model used in the study comprises six encoders. In the ATS procedure, the numbers of tokens kept in each layer were 256, 128, 64, 32, 16, and 8, which was the default setting from the original paper of the ATS. These parameters were obtained based on empirical results. It is noted that we tried a variety of token sample numbers in addition to the default setting and did not observe a significant difference in results, which was because of the fact that the sampling strategy of the ATS ensures that the model focuses on key regions, but does not completely discard the information of some outlier data, so it can adjust the pattern extraction ability of the model for different types of data according to the input.

**TABLE 3:** Training setting.

Hyperparameter	Value
Learning rate	2e-5
Eps	1e-8
Batch size	64
Epochs	300
Input image size	256 × 256
ATS # tokens	[256, 128, 64, 32, 16, 8]

Both datasets are split into training, validation, and test sets in the ratio of 7:1:2. In addition, the training set is further split in the ratio of 8:2; 80% of the data in the training set participate in the supervised training to learn an ATS-ViT model, and the rest 20% are treated as unlabeled data used for CT.

### 4.4 Results

Table 4 presents a performance comparison between the proposed method and the chosen baselines. Also, an ablation study has been conducted to evaluate the efficacy of the ATS and CT. Specifically, we used the ViT as a base model and added the ATS and CT to form the ViT + ATS model and the CT + ViT + ATS model. For each evaluated model, four metrics defined in Section 3.1 have been reported, including Acc, Pre, Rec, and F1. We provide the result interpretation as follows.

• It is observed that the CNN models, namely, VGG19, ResNet101, and DenseNet201, can achieve similar performance compared with the ViT base model. In particular, ResNet101 presents the highest Acc (95.59%) and F1 (94.76%) among the four baselines.
• The ViT base model does not perform better in our experiments than the CNN models. In the original study on the ViT, it has been validated to outperform the CNN models on several image classification tasks such as ImageNet (Deng et al., 2009). In our experiment, the ViT achieves an Acc of 93.38% and an F1 of 93.43%, ranked the third and second places among the four baselines. The reason why the ViT does not outperform all CNN models may be because of the training configuration or the hyperparameter setting that has not been sufficiently optimized.
• The addition of the ATS to the ViT has improved the Acc and F1 by 1.07 and 1.04%, respectively. However, the ViT + ATS is still not as good as ResNet101. The performance gain is mainly due to the sampling strategy that can effectively select a subset of tokens that contribute the most to the classification task.
• Our best model, namely, CT + ViT + ATS, achieves the best results on all four metrics with 95.29% Acc, 96.29% Pre, 96.01% Rec, and 95.15% F1, outperforming the second-best scores by 0.34, 2, 0.78, and 1.39%, respectively. Compared with the Vit + ATS model, the four scores have improved by 0.84, 2, 1.23, and 1.86%. The performance gains are mainly due to the training procedure that combines both supervised and unsupervised training so that the model can experience more diversified samples via data augmentation during consistency training.

**TABLE 4:** Results on BUSI.

Model	Acc	Pre	Rec	F1
VGG19	93.02	92.3	92.07	92.19
ResNet101	94.95	94.29	95.23	94.76
DenseNet201	93.62	92.88	93.71	93.29
ViT	93.38	93.02	93.37	93.43
ViT + ATS	94.45	94.29	94.78	94.47
CT + ViT + ATS (ours)	**95.29**	**96.29**	**96.01**	**96.15**

The highest scores of each metric are in bold.

Table 5 shows the results of the validated models on BreakHis. The same set of models has been evaluated, and the results are similar to the ones on BUSI. We highlight the observations as follows.

• Among the four baseline models, DenseNet201 shows the highest Acc of 97.42%, while VGG19 presents the highest F1 of 96.16%. The ViT base model posts an Acc of 95.68% and an F1 of 95.69%, ranked the third and second places, respectively. Again, the ViT does not stand out on this classification task.
• The addition of the ATS improves the Acc and F1 by 1.3 and 0.57%, respectively, lifting the model to the top place in F1 (96.26), with CT + ViT + ATS excluded. This improvement shows that the ATS can effectively locate the image tokens with the most informative parts, allowing the model to learn more distinguishable patterns to boost accuracy. The result shows that the ATS presents the desired effect and has been consistent across both classification tasks.
• CT + ViT + ATS, on the other hand, achieves the best performance for all four metrics with an Acc of 98.12%, a Pre of 98.17%, a Rec of 98.65%, and an F1 of 98.41%. This result shows that CT can bring consistent performance boost on both datasets and is a promising strategy to improve a model’s generalization ability.

**TABLE 5:** Results on BreakHis.

Model	Acc	Pre	Rec	F1
VGG19	96.41	96.45	95.88	96.16
ResNet101	95.53	95.54	94.38	94.96
DenseNet201	97.42	93.98	97.89	95.6
ViT	95.68	95.67	95.7	95.69
ViT + ATS	96.98	96.85	95.68	96.26
CT + ViT + ATS (ours)	**98.12**	**98.17**	**98.65**	**98.41**

The highest scores of each metric are in bold.

**FIGURE 5:**
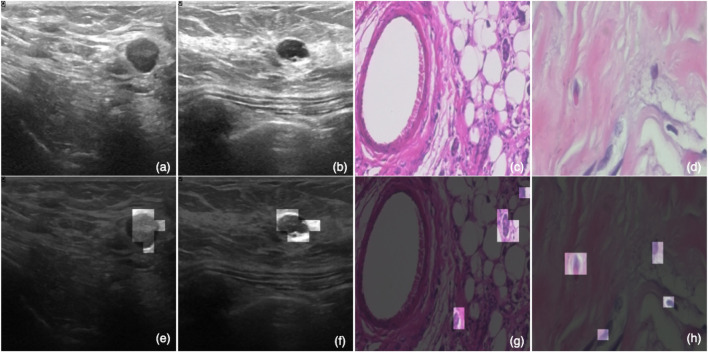
Visualized effect of the ATS. Subfigures **(A,B)** are ultrasound images; and **(C,D)** are histopathology samples. Meanwhile, **(E–H)** are the same images as **(A–D)** with the eight most significant tokens (image patches) kept for each image.

Figure 5 shows the effect of the ATS on the four samples, with two from each dataset. In this, Figures 5A,B are ultrasound images; and Figures 5C,D are histopathology samples. Meanwhile, Figures 5E–H are the same images as Figures 5A–D with the eight most significant tokens (image patches) kept for each image. These eight tokens are obtained from the last transformer block, which is closer to the detection head, and thus, is more expressive for the classification result. It is observed that these tokens can accurately identify the regions of interest that are more indicative of the actual classes. Instead of looking at the whole image, an ATS-enabled model can reduce the amount of global information and pinpoint the most critical areas that contribute the most to the prediction results, which explains the effectiveness of the ATS.

## 5 Discussion

This study presents CT + ViT + ATS, a ViT model trained *via* CT and boosted via ATS. The proposed model has been validated on two BC imaging datasets and shown superior performance compared to three representative CNN baseline models. The results have demonstrated the efficacy of both the ATS and CT. The former allows the learning algorithm to identify the regions of interest that provide significant patterns for the classification task, and the latter unifies both supervised and unsupervised training to improve the generalization ability of the model. The proposed model, with the validated results, can serve as a credible benchmark for future research.

There are several notable findings from this study. Our experimental results show that the original ViT model does not present superior performance compared to its CNN competitors. On the BUSI dataset, the ViT is on a par with the CNN models, whereas on the BreakHis dataset, the ViT is slightly worse but still comparable. This could be because of the BC detection task, in which the images may contain subtle patterns hard to capture even with the self-attention mechanism used by the ViT. To discover these subtle patterns and improve detection accuracy, we adopt the ATS and CT as two boosting modules, which turn out to be effective. The gains, in Acc and F1, brought by the ATS and CT, have been notable and consistent on both datasets. Although the ATS was originally developed to reduce computational costs, we demonstrate that it also improves the detection accuracy since the model is encouraged to focus more on the critical image tokens and learn more subtle patterns. CT, on the other hand, exploits the existing training resources *via* a weakly-supervised training paradigm that effectively improves the robustness of the model. The two boosting modules refine the original ViT in three aspects: model, data, and training procedure. These joint efforts have been consistent for our task and have the potential to be used for other biomedical computer vision tasks.

The proposed CT + ViT + ATS method can be a core functional module of a CAD system for BC detection. It offers two merits. First, the ATS component allows the system to highlight the most informative image patches, which can help physicians quickly pinpoint the critical areas for precise and personalized diagnosis. Second, the backend of the CAD system can be easily modified to be a continuous learning system once new images are available. Since CT is semi-supervised, only a portion of the newly added data needs to be labeled, significantly reducing the labor cost for annotation.

The proposed method can be extended in the following directions. First, we mainly compared CNN models and the ViT, while an ensemble of the two or feature-level aggregation can be another model design option that may bring together the strengths of both neural architectures. Given that the underlying designs of the CNN and the ViT are fundamentally different, the former adopts multiple filters to capture multi-scale features, while the latter explores semantic relations between each pair of tokens; a combination of the two could present superior performance compared to any single model. Second, a generative model such as a generative adversarial network (GAN) can be used to perform data augmentation in CT. Since a GAN captures the distribution of images belonging to a class, a well-trained GAN can generate synthetic images that look similar to real ones. These generated images can enhance the quantity and diversity of the training samples during CT, potentially leading to a more robust model. Lastly, the proposed method can be applied to a wider range of BC imaging datasets with additional image modalities such as X-ray, MRI, and thermography that are not considered in this study. It would be interesting to evaluate the proposed method on a multi-modal BC imaging dataset that offers multi-dimensional feature representations.

## Data Availability

The datasets presented in this study can be found in online repositories. The names of the repository/repositories and accession number(s) can be found at: https://www.kaggle.com/datasets/aryashah2k/breast-ultrasound-images-dataset (accessed on 20 November 2021) and http://web.inf.ufpr.br/vri/breast-cancer-database (accessed on 25 November 2021).
